# Comparison of rectal swabs and faeces for real-time PCR detection of enteric agents in Rwandan children with gastroenteritis

**DOI:** 10.1186/1471-2334-13-447

**Published:** 2013-09-27

**Authors:** Jean-Claude Kabayiza, Maria E Andersson, Christina Welinder-Olsson, Tomas Bergström, Gregoire Muhirwa, Magnus Lindh

**Affiliations:** 1 Department of Paediatrics, National University of Rwanda, Butare, Rwanda; 2 Department of Clinical Biology, National University of Rwanda, Butare, Rwanda; 3 Department of Infectious Diseases, University of Gothenburg, Gothenburg, Sweden

## Abstract

**Background:**

Molecular diagnostics have emerged as an efficient and feasible alternative for broad detection of pathogens in faeces. However, collection of stool samples is often impractical in both clinical work and in epidemiology studies. The aim of this study was to investigate the diagnostic performance of rectal swabs as compared with traditional faeces samples for detection of enteric agents by PCR.

**Method:**

Three hundred twenty-six pairs of rectal swab and stool samples, obtained from Rwandan children aged 0.5-4.99 years, with or without diarrhoea, were analysed by multiple real-time PCR amplifying 3 viral, 6 bacterial and one protozoan target.

**Results:**

For all agents there was a significant correlation (R^2^ 0.31-0.85) between Ct values in faeces and rectal swabs. For most agents the Ct values, a marker for target concentration, were significantly lower (by 1–3 cycles) in faeces, indicating pathogen content up to ten times higher than in rectal swabs. Despite this, there was no significant difference in detection rate between faeces and rectal swabs for any agent, reflecting that pathogen concentration was far above the limit of detection in the majority of cases.

**Conclusion:**

The similar detection rates and the Ct value correlations as compared with traditional faeces samples indicate that rectal swabs are accurate for real-time PCR-based identification of enteric agents and may be used also for quantitative estimation of pathogen load.

## Background

Acute gastroenteritis is an important cause of morbidity and mortality in children in developing countries. It can be caused by a wide range of microbial agents, which may be identified by various techniques such as direct microscopy, antigen detection, culture, electron microscopy and nucleic acid amplification. Detection of viruses is now best performed by molecular assays [1], although antigen detection is still widely used for rotavirus. Bacterial diagnostics still mainly relies upon culture, but detection by PCR is established for *Clostridium difficile*[2,3], and may provide better sensitivity also for diagnostics of other bacteria [4-7]. Culture of bacteria remains important because it provides resistance profile, whereas a main advantage of molecular techniques is that they give more rapid results and allow combined detection of viral and bacterial agents. Such broad multiplex PCR methods are likely to improve routine diagnostics, and their application in research will expand our knowledge about the epidemiology of diarrhoeal diseases [1,8].

Rectal swabs are advantageous over traditional stool samples because collection, transport and further handling becomes more feasible, and indeed swabs have been used for decades as an alternative to faeces for bacterial culture [9]. Some previous studies have used rectal swabs for molecular testing of pathogens in faeces [2,10-14], but a focused comparison of rectal swabs and faeces has been performed only for a few agents and on limited number of samples. Thus, additional studies are required in order to further document the utility of rectal swab sampling for molecular diagnostics of diarrhoeal diseases. In the present study, real-time PCR for a range of pathogens was used to compare rectal swabs and stool samples for diagnostics of enteric infections in children.

## Methods

### Patients

The samples were obtained from children 0.5-4.99 years of age, who were seeking medical care at the Departments of Paediatrics at the University Hospitals in Kigali and Butare, Rwanda, or at 2 district hospitals and 2 health centres in these two cities. The children participated in a study of acute gastroenteritis between November 2009 and May 2011, including 650 patients (children with >3 loose stools per day for less than 96 hours, with or without vomiting or fever) and 160 healthy controls (children living in the same geographic area, in the same age span, and without diarrhoea during the last 14 days). When possible, both a rectal swab and a faeces sample was obtained, and the present study includes all 326 such paired samples.

### Stools sample collection

From each patient stool was collected as both a rectal swab (Copan Regular Flocked Swab 502CS01, Copan Italia Spa, Brescia, Italy) in a tube with 1 mL of sterile saline, and as 2 mL of faeces. Rectal swabs were obtained by inserting the swab into the rectum and rotating it a few times before retraction. The samples were kept at below +8°C for a few hours and were sent to a local laboratory for storage at -80°C until transport (without thawing) to the Department of Clinical Virology at Gothenburg University, Sweden where molecular testing was performed.

### Microbial agents and target sequences

The targets for real-time PCR are described in Table 1. The amplified regions of rotavirus, norovirus and adenovirus were located to conserved genomic regions [15-17], and these assays have been used in our diagnostic laboratory several years. The bacterial PCRs were developed by guidance from available publications [18-24], usually by adapting a traditional PCR method to real-time PCR (when this study was planned, suitable real-time PCR assays were lacking for most non-viral targets). Thus, established target regions were used, and primers and probes were designed with the aim of obtaining similar melting temperatures (≈58-60°C for primers, ≈68-70°C for probes). For enterotoxin-producing *Escherichia coli* (ETEC), both heat labile toxin (*eltB*) and heat stable toxin (*estA*) coding regions were targeted. *E. coli* genes coding for enteroaggregative factor (*eae*) and bundle-forming pilus (*bfpA*) are important for adherence of *E. coli* to the intestinal mucosa, and are considered to be markers for enteropathogenic *E. coli* (EPEC), in particular when both are present. *Shigella* were identified by amplification of the invasion plasmid antigen H gene (*ipaH*, which also may be present in entero-invasive *E. coli*, EIEC); *Campylobacter jejuni* by the fibronectin-binding protein (cadF) gene; *Cryptosporidium parvum/hominis* by the oocyst wall protein (OWP) gene. Sufficient amplification efficiencies were documented for each bacterial real-time PCR by analysing serial dilutions of pUC57 plasmids carrying synthetic target inserts. The accuracy of the bacterial assays was further evaluated by analysing *ETEC* and *EHEC* strains identified at the Department of Bacteriology, Sahlgrenska University Hospital, reference strains *Campylobacter jejuni* and *Shigella flexneri* from the culture collection of the University of Gothenburg (CCUG), and *Cryptosporidium parvum* DNA obtained from the American Type Culture Collection (ATCC).

**Table 1:** Primers and probes targeting RNA or DNA of diarrheagenic agents

**Agent**	**Forward primer**	**Reverse primer**	**Probe**	**Target gene**	**Ref**
Norovirus GG2	TGGAYTTTTAYGTGCCCAG	CGACGCCATCTTCATTCAC	AGCCAGATTGCGATCGCCC	Polymerase-capsid junction	[16]
Rotavirus^a^	AACCATCTACACATGACCCTCTATGA	GGTCACATAACGCCCCTATAGC	CAATAGTTAAAAGCTAACACTGTCAAA	Non-strutural protein 3	[17]
	AACCATCTTCACGTAACCCTCTATGA				
Adenovirus	GCCACGGTGGGGTTTCTAAACTT	GCCCCAGTGGTCTTACATGCACATC	TGCACCAGACCCGGGCTCAGGTACTCCGA	Hexon	[15]
*Campylobacter jejuni*	ATGCAAACCATAATTGGGTTTCAAC	CGAGTATCAGCAACTTCTTCTACAGCT	TTGCCACCAAAACCAAAACT	Fibronectin-binding protein (cadF)	[18]
*ETEC-estA*	AAGCATGAATAGTAGCAATTACTGCT	TTAATAGCACCCGGTACAAGCA	AACAACACAATTCAC	Heat-stable enterotoxin	[19]
*ETEC-eltB*	TCCGGCAGAGGATGGTTACA	CCAGGGTTCTTCTCTCCAAGC	AGCAGGTTTCCCACCGGATCACC	Heat-labile enterotoxin	[20]
*E. coli eae*	ACATGACCGATGACAAGGCA	CGCGACTGAAGCTGGCTAC	TCGCCGCCTGTTGTGCCG	Enteroaggregative factor	[21]
*E. coli bfpA*	GGTCTGTCTTTGATTGAATCTGCA	GCAGACTGGTAGTAAAACATCACACC	GCGCTTGCTGCCACCGTTACCG	Bundle-forming pilus	[22]
*Shigella spp*	ACCGGCGCTCTGCTCTC	GCAATGTCCTCCAGAATTTCG	CTGGGCAGGGAAATGTTCCGCC	Invasion plasmid antigen H	[23]
*Cryptosporidium parvum/hominis*	CAAATTGATACCGTTTGTCCTTCTG	TGGTGCCATACATTGTTGTCCT	TGTCCTCCTGGATTCA	Oocyst wall protein (OWP)	[24]

^a^Two forward primers were used for rotavirus.

GG2, genogroup 2, ETEC, enterotoxinogenic *E. coli.*

### Sample preparation and nucleic acid extraction

Approximately 250 μL of faeces were dissolved in 4.5 mL of saline and centrifuged 5 minutes at 750 × g. Then, 250 μL of the dissolved faeces or 250 μL of the rectal swab were mixed with 2 mL of lysis buffer, and this volume was used for extraction of total nucleic acid in an EasyMag instrument (Biomerieux, Marcy l’Étoile, France). The nucleic acids were eluted in 110 μL volume, and 5 μL of this were used for real-time PCR. These procedures correspond to an approximate dilution of faeces to 1:10 prior to PCR. The dilution of rectal swab samples is uncertain because it depends on the specimen volume contained in the swab after retraction from rectum. Thus, even though the swab may collect up to 140 μL (corresponding to a 1:4 dilution prior to PCR), the volume left after rectal sampling may be considerably less, possibly only 10–20 μL corresponding to 1:50 dilution or more). The dilution has the advantage that it reduces the potential impact of inhibitors. We evaluated the whether there was any inhibition of PCR by spiking 24 faeces samples and 24 rectal swabs with seal herpes virus (PhHV-1) prior to extraction and analysis by PhHV-1 real-time PCR [25].

### Real-time PCR

Amplification in an ABI 7900 instrument (Applied Biosystems, Foster City, CA) in 7 parallel 20 μL-reactions containing oligonucleotides described in Table 1, and Taqman Fast Virus 1-step Mastermix (ABI, for RNA targets) or Universal Mastermix (ABI, for DNA targets). A two-step amplification (15 sec 95°C, 60 sec 56°C) was run for 45 cycles after an initial 10 min denaturation 95°C and 30 min reverse transcription at 46°C. In each run plasmids containing the target regions for all agents were amplified in parallel with patient specimens to verify the performance of each target PCR (mastermix control).

### Analysis of human gene content in faeces and rectal swabs

In order to evaluate to what extent faeces and rectal swabs differed in regard to content of human cells we analysed 24 faeces and 24 rectal swab samples by real-time PCR targeting betaglobin, a human gene.

### Ethical committee approval

The study was approved by the regional ethics board in Gothenburg and the ethical committee at National University of Rwanda. An informed consent was obtained from carers of children enrolled in the study.

### Statistics

Statistical analysis was performed using the Statview software (SAS). Ct (threshold cycle) values in faeces and rectal swabs were compared by paired *t* test (for samples reactive in both specimen types). Ct values in faeces and rectal swabs were also compared by Mann–Whitney *U* test, including also test negative samples given an arbitrary Ct value of 45. The proportions reactive (detected or not detected) in faeces and rectal swab were compared by Fisher’s exact test. Ct values obtained by real-time PCR of faeces and rectal swab samples were compared by Pearson’s correlation analysis.

## Results

### Detections rates in faeces and rectal swabs

As shown in Table 2, the sensitivity in terms of detection rate was similar for faeces as compared with rectal swabs, with the exception of *E. coli* with an *eltB* gene, which among patients tended to be more often detected in faeces (38% vs. 29%, P = 0.06). The highest rate of detection was observed for adenovirus (55% in controls), but rates above 20% were also observed for several other agents.

**Table 2:** Detection rates by real-time PCR of 326 paired faeces and rectal swab samples in samples from subjects with or without diarrhoea

	**Diarrhoea (n = 207)**					**No diarrhoea (n = 119)**				
	**Faeces**		**Rectal swab**		**P value**	**Faeces**		**Rectal swab**		**P value**
*ETEC-eltB*	38%	(78)	29%	(60)	0.06	47%	(56)	42%	(50)	>0.30
*ETEC-estA*	16%	(34)	15%	(31)	>0.30	10%	(12)	13%	(16)	>0.30
*E. coli bfpA*	21%	(44)	17%	(35)	0.26	7.6%	(9)	9.2%	(11)	>0.30
*E. coli eae*	22%	(45)	22%	(45)	>0.30	23%	(27)	25%	(30)	>0.30
*Shigella ipaH*	22%	(46)	19%	(39)	>0.30	17%	(20)	22%	(26)	>0.30
*Campylobacter*	20%	(42)	20%	(42)	>0.30	22%	(26)	20%	(24)	>0.30
*Cryptosporidium*	9.7%	(20)	9.2%	(19)	>0.30	5.0%	(6)	6.7%	(8)	>0.30
Norovirus GG2	8.7%	(18)	8.2%	(17)	>0.30	4.2%	(5)	3.4%	(4)	>0.30
Rotavirus	23%	(48)	23%	(48)	>0.30	0%	(0)	0.9%	(1)	>0.30
Adenovirus	13%	(26)	16%	(33)	>0.30	50%	(59)	55%	(65)	>0.30

Number of cases in paranthesis.

ETEC, enterotoxinogenic *E. coli*. GG2, genogroup 2.

Adenovirus was analysed in 193 of the 326 samples.

If reactivity in either faeces or rectal swabs was considered as gold standard the sensitivities ranged from 73% to 91% in faeces and from 75% to 90% in rectal swabs, with Kappa values ranging from 0.452 to 0.821, as shown in Table 3.

**Table 3:** Sensitivities and agreements of PCR detection between faeces and rectal swabs

	**Reactive in faeces or rectal swab**	**Reactive in faeces**	**Faeces sensitivity**^**a**^	**Reactive in rectal swab**	**Rectal swab sensitivity**^**b**^	**Reactive in faeces *and *rectal swab**	**Discordant samples**^**c**^	**Kappa value**
*ETEC-eltB*	147	134	91%	110	75%	97	50	0.674
*ETEC-estA*	56	46	82%	47	84%	37	19	0.762
*E. coli bfpA*	61	53	87%	46	75%	38	23	0.726
*E. coli eae*	91	72	79%	75	82%	56	35	0.692
*Shigella ipaH*	77	66	86%	65	84%	54	23	0.780
*Campylobacter*	76	68	89%	65	86%	57	19	0.821
*Cryptosporidium*	31	25	81%	28	90%	22	9	0.815
Rotavirus	60	48	80%	49	82%	37	23	0.721
Norovirus GG2	27	23	85%	21	78%	17	10	0.756
Adenovirus	122	85	73%	95	84%	65	53	0.452

^a^Reactive in faeces/reactive in either faeces or rectal swab.

^b^Reactive in rectal swab/reactive in either faeces or rectal swab.

^c^Reactive in rectal swab or faeces, not in both.

ETEC, enterotoxinogenic *E. coli*. GG2, genogroup 2.

Adenovirus was analysed in 193 of the 326 samples.

### Ct values in faeces and rectal swabs

The median Ct values ranged from ≈ 20 cycles for rotavirus to ≈ 35 for *Cryptosporidium*, as shown in Table 4. The Ct values for discordant samples (reactive in only one specimen type) were higher (i.e. pathogen load was lower) than for those reactive in both specimens, and typically were above 35 cycles.

**Table 4:** Median Ct values and Ct value differences by real-time PCR

	**Median Ct value**^**a**^		**dCt**^**b**^**, mean (SE)**	**P value**^**c**^
	**Rectal swab**	**Faeces**		
*ETEC-eltB*	32.0/37.5	28.5/37.4	2.28 (0.48)	<0.0001
*ETEC-estA*	27.7/36.2	25.8/40.1	2.99 (0.48)	<0.0001
*E. coli bfpA*	27.6/34.2	26.9/37.5	2.10 (0.49)	<0.0001
*E. coli eae*	34.8/38.1	33.5/38.5	0.85 (0.47)	0.0743
*Shigella ipaH*	29.4/37.8	28.1/36.7	0.91 (0.58)	0.1245
*Campylobacter*	32.6/35.8	33.2/37.4	-0.002 (0.59)	1.00
*Cryptosporidium*	36.2/39.9	34.1/40.9	2.23 (0.76)	0.008
Rotavirus	22.5/41.2	20.9/36.2	2.25 (0.61)	0.0008
Norovirus GG2	30.3/38.8	29.3/33.8	1.45 (0.78)	0.08
Adenovirus	34.0/39.7	35.9/39.9	-0.48 (0.39)	0.21

^a^Concordant/discordant, i.e. reactive in both rectal swab and faeces/reactive in either rectal swab or faeces. Negative results excluded.

^b^dCt = Ct in rectal swab–Ct in faeces. For samples reactive in both rectal swab and faeces.

^c^By paired *t* test.

ETEC, enterotoxinogenic *E. coli*. GG2, genogroup 2.

**Figure 1:**
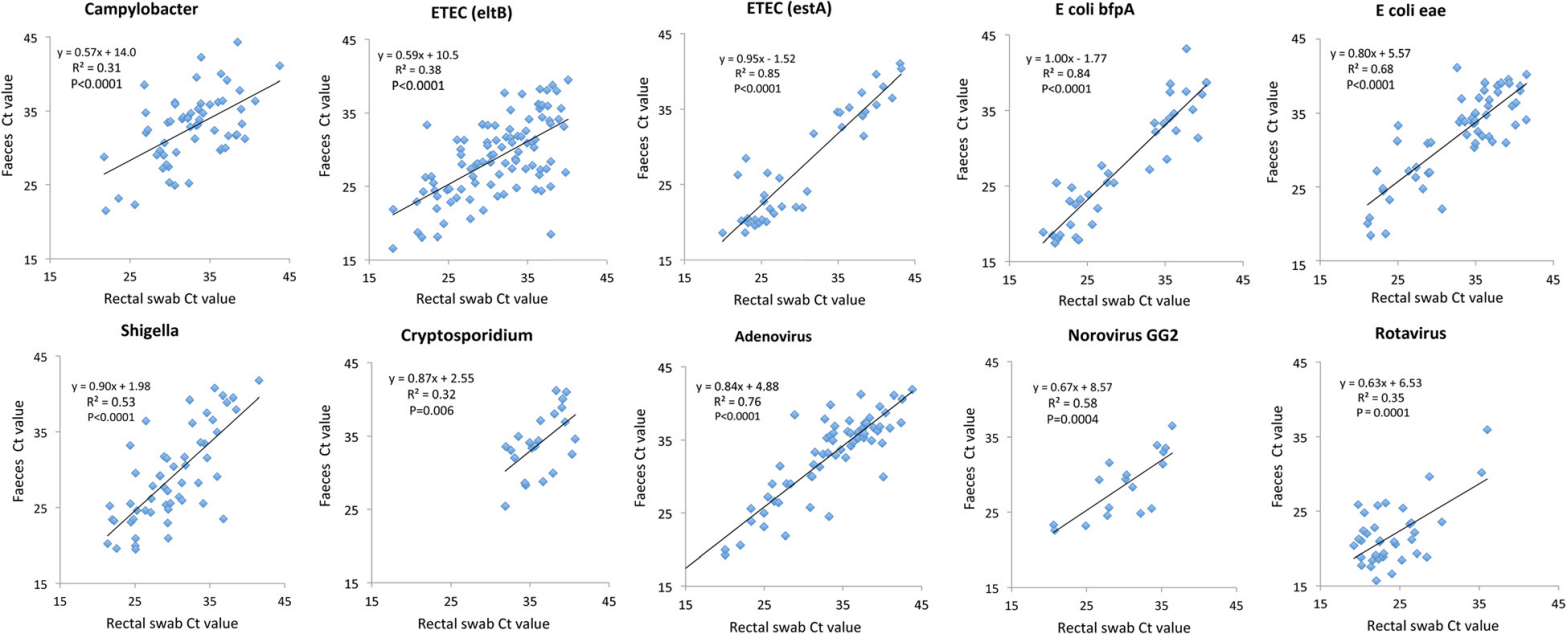
Correlation between Ct values observed by real-time PCR analysis of rectal swabs and faeces on samples that were reactive in both specimen types.

For samples that were reactive in both rectal swabs and faeces there was a significant correlation between Ct values for all agents (R^2^ 0.31-0.85, Figure 1). For most targets the Ct values were significantly higher (1–3 cycles) in rectal swab than in faeces (Table 4). Of note this difference was not observed for adenovirus and *Campylobacter*, and as shown in Table 5, it was confined to or more pronounced in samples from children with diarrhoea as compared with without diarrhoea.

**Table 5:** Comparison of Ct values in faeces and rectal swabs in children with or without diarrhoea

	**Diarrhoea (n = 207)**			**No diarrhoea (n = 119)**		
	**Mean Ct difference**^**a**^	**n**	**P value**	**Mean Ct difference**^**a**^	**n**	**P value**
*ETEC-eltB*	3.61	47	<0.0001	1.03	50	0.09
ETEC-est A	3.11	24	<0.0001	2.76	13	0.02
*E. coli bfpA*	2.27	28	0.0002	1.63	10	0.20
*E. coli eae*	2.40	30	0.0001	-0.93	26	0.1662
*Shigella*	2.41	34	<0.0004	-1.64	20	0.1008
*Campylobacter*	-0.61	30	0.50	-0.69	27	0.36
*Cryptosporidium*	3.09	16	0.002	-0.06	6	0.96
Rotavirus	2.25	37	0.0008		0	
Norovirus GG2	2.21	12	0.04	-0.39	5	0.72
Adenovirus	0.43	12	0.71	-0.69	53	0.087

^a^Ct value in rectal swab–Ct value in faeces.

Comparison of subset of cases that were reactive in both faeces and rectal swabs.

P values by paired *t* test.

GG2, genogroup 2.

### Inhibition and human cell content

Analysis of spiked seal herpes virus by real-time PCR did not show inhibition in any sample, indicated by a small degree of variation of the PhHV-1 DNA Ct values, ranging between 25.9 and 27.7 cycles (mean 26.8).

Analysis of betaglobin showed that the content of human cells differed significantly. In faeces 12 out of 24 samples had detectable betaglobin DNA and the Ct values in positive cases were high (mean 38.2). In contrast, betaglobin was detected in all rectal swab samples with Ct values ranging from 25 to 35 (mean 30), indicating relatively high cell content.

## Discussion

The results of the present study of 326 paired specimens from Rwandan children with or without diarrhoea demonstrate a good correlation between results obtained by real-time PCR analysis of faeces and rectal swabs, both in terms of rates of detection and Ct values (a quantitative parameter that reflects the target concentration). This agrees with previous reports of good performance of rectal swabs for PCR detection of diarrhoeagenic viruses [10], *Clostridium difficile*[2] and *Enterobacter*[14]. As compared with these studies, the present investigation was performed on a larger set of samples, a broader range of agents and in a more difficult setting, comprising sampling of small children at several health care units in a developing country. The performance of PCR on rectal swabs was good, with equal rates of detection (i.e. clinical sensitivity) as compared with faeces for all but one of the pathogens. In samples with disagreement, i.e. when PCR was positive in only either of rectal swab or faeces, the Ct value was usually above 35 cycles, indicating a low target concentration. Overall, these finding support that rectal swabs are adequate for collecting stool specimens aimed for detection by molecular techniques.

The Ct value of real-time PCR represents an estimate of the concentration of the targeted agents in stool. In the present study, rectal swab Ct values correlated significantly with faeces Ct values for all targets, with R^2^ ranging from 0.31 to 0.85. This is important because it indicates that rectal swabs may be used also to study pathogen load. Such quantitative information may become critical for understanding the aetiology of gastroenteritis, and in epidemiologic studies the convenience of collecting specimen by rectal swabs rather than faeces would be a significant advantage. High rates of detection were observed in both patients and healthy children, indicating that the mere detection of pathogen DNA may be difficult to interpret. In future studies we plan to compare Ct values in patients and healthy controls with the aim of identifying cut-off values that distinguish symptomatic and asymptomatic infections.

Despite the good Ct value correlation, some cases were reactive in only one specimen type or differed considerably in Ct value between faeces and rectal swab. Some of these discordant samples were reactive in rectal swab but not in faeces, which seems difficult to understand. Maybe, in at least some of the cases, the pathogen was present in the rectal mucosa rather than in faeces, within or attached to epithelial cells. This possibility was supported by the finding that rectal swabs, but not faeces, contained significant amounts of human betaglobin DNA (indicating a high human cell content). It was less surprising that some rectal swabs were test negative despite reactivity in faeces, because the amount of faeces in a swab may be small and sometimes almost invisible (if diarrhoea is watery). An insufficient sample collection may occur if the swab is not inserted properly into the rectum, but this should have been rare since sampling was performed by study nurses that had received instruction about the procedure. Alternatively, a significant part of the sample may be lost during retraction of the swab from rectum. This might explain the finding that Ct values for rectal swabs on average were 2–3 cycles higher than for faeces, a difference that roughly corresponds to a ten times lower amount of pathogen content in PCR performed on rectal swab as compared with faeces. As mentioned above this did not result in any difference in crude detection rate, because pathogen load was far above the detection limit in most cases. However, if Ct values prove clinically useful, such a 2–3 cycles difference between faeces and rectal swabs will have to be taken into account when the quantitative information is interpreted.

These findings underline that standardisation will be of importance for quantitative applications. In a recent study it was suggested that normalisation of the pathogen Ct value to the Ct value obtained by real-time PCR targeting 16S may allow a more accurate estimate of pathogen load in faeces [14]. By such a procedure the authors observed a good correlation between bacterial gene quantification in faeces and rectal swabs. However, the degree of correlation reported by this strategy (R^2^ = 0.55) was similar to what was observed in the present study comparing Ct values without normalisation (R^2^ = 0.31-0.85). This indicates that the value of normalisation and how it should be performed requires further study.

## Conclusions

The similar detection rates and the Ct value correlations as compared with traditional faeces samples indicate that rectal swabs are accurate for real-time PCR-based identification of a wide range of enteric pathogens and may be used also for quantitative estimation of pathogen load. Rectal swabs are advantageous because they can be obtained without delay in outpatient settings, and also significantly simplify sample collection from patients staying in the hospital. This sampling mode may facilitate clinical care and studies of diarrhoeal diseases in children in developing countries.

## Competing interests

The authors declare that they have no competing interests.

## Authors’ contributions

JCK planned the study, conducted the field studies and sample collection and drafted the manuscript. MA developed and carried out the real-time PCR and participated in the data analysis. CWO participated in the development of real-time PCR. GM participated in planning the field studies. TB participated in analysing the data. ML planned and coordinated the study, designed the real-time PCR assays, analysed the data and finalised the manuscript. All authors read and approved the final manuscript.

## Pre-publication history

The pre-publication history for this paper can be accessed here:

http://www.biomedcentral.com/1471-2334/13/447/prepub

## References

[B1] Wolffs PFG Bruggeman CA Van Well GTJ Van Loo IHM Replacing traditional diagnostics of fecal viral pathogens by a comprehensive panel of real-time PCRs J Clin Microbiol 2011 49 1926 1931 10.1128/JCM.01925-10 21430103 PMC3122640

[B2] Shakir FA Thompson D Marlar R Ali T A novel use of rectal swab to test for clostridium difficile infection by real-time PCR Am J Gastroenterol 2012 107 1444 1445 10.1038/ajg.2012.162 22951884

[B3] Kundrapu S Sunkesula VC Jury LA Sethi AK Donskey CJ Utility of perirectal swab specimens for diagnosis of clostridium difficile infection Clin Infect Dis 2012 55 1527 1530 10.1093/cid/cis707 22911648

[B4] Wang S-M Ma J-C Hao Z-Y Zhang Z-Y Mason C Sethabutr O Von Seidlein L Wang X-Y Xu Z-Y Surveillance of shigellosis by real-time PCR suggests underestimation of shigellosis prevalence by culture-based methods in a population of rural China J Infect 2010 61 471 475 10.1016/j.jinf.2010.10.004 20951728

[B5] De Boer RF Ott A Kesztyus B Kooistra-Smid AM Improved detection of five major gastrointestinal pathogens by use of a molecular screening approach J Clin Microbiol 2010 48 4140 4146 10.1128/JCM.01124-10 20861334 PMC3020836

[B6] Iijima Y Asako NT Aihara M Hayashi K Improvement in the detection rate of diarrhoeagenic bacteria in human stool specimens by a rapid real-time PCR assay J Med Microbiol 2004 53 617 622 10.1099/jmm.0.45607-0 15184531

[B7] Schuurman T De Boer RF Van Zanten E Van Slochteren KR Scheper HR Dijk-Alberts BG Moller AV Kooistra-Smid AM Feasibility of a molecular screening method for detection of Salmonella enterica and Campylobacter jejuni in a routine community-based clinical microbiology laboratory J Clin Microbiol 2007 45 3692 3700 10.1128/JCM.00896-07 17804656 PMC2168500

[B8] Liu J Gratz J Amour C Kibiki G Becker S Janaki L Verweij JJ Taniuchi M Sobuz SU Haque R A laboratory-developed TaqMan array card for simultaneous detection of 19 enteropathogens J Clin Microbiol 2013 51 472 480 10.1128/JCM.02658-12 23175269 PMC3553916

[B9] McFarland LV Coyle MB Kremer WH Stamm WE Rectal swab cultures for Clostridium difficile surveillance studies J Clin Microbiol 1987 25 2241 2242 3693551 10.1128/jcm.25.11.2241-2242.1987 PMC269456

[B10] Gustavsson L Westin J Andersson LM Lindh M Rectal swabs can be used for diagnosis of viral gastroenteritis with a multiple real-time PCR assay J Clin Virol 2011 51 279 282 10.1016/j.jcv.2011.05.025 21683649

[B11] Nakanishi K Tsugawa T Honma S Nakata S Tatsumi M Yoto Y Tsutsumi H Detection of enteric viruses in rectal swabs from children with acute gastroenteritis attending the pediatric outpatient clinics in Sapporo, Japan J Clin Virol 2009 46 94 97 10.1016/j.jcv.2009.06.014 19608459

[B12] Lautenbach E Harris AD Perencevich EN Nachamkin I Tolomeo P Metlay JP Test characteristics of perirectal and rectal swab compared to stool sample for detection of fluoroquinolone-resistant Escherichia coli in the gastrointestinal tract Antimicrob Agents Chemother 2005 49 798 800 10.1128/AAC.49.2.798-800.2005 15673772 PMC547369

[B13] Vu DT Sethabutr O Von Seidlein L Tran VT Do GC Bui TC Le HT Lee H Houng HS Hale TL Detection of Shigella by a PCR assay targeting the ipaH gene suggests increased prevalence of shigellosis in Nha Trang, Vietnam J Clin Microbiol 2004 42 2031 2035 10.1128/JCM.42.5.2031-2035.2004 15131166 PMC404673

[B14] Lerner A Romano J Chmelnitsky I Navon-Venezia S Edgar R Carmeli Y Rectal swabs are suitable for quantifying the carriage load of KPC-producing carbapenem-resistant Enterobacteriaceae Antimicrob Agents Chemother 2013 57 1474 1479 10.1128/AAC.01275-12 23295937 PMC3591867

[B15] Heim A Ebnet C Harste G Pring-Akerblom P Rapid and quantitative detection of human adenovirus DNA by real-time PCR J Med Virol 2003 70 228 239 10.1002/jmv.10382 12696109

[B16] Nenonen NP Hannoun C Olsson MB Bergstrom T Molecular analysis of an oyster-related norovirus outbreak J Clin Virol 2009 45 105 108 10.1016/j.jcv.2009.04.011 19451026

[B17] Pang XL Lee B Boroumand N Leblanc B Preiksaitis JK Yu Ip CC Increased detection of rotavirus using a real time reverse transcription-polymerase chain reaction (RT-PCR) assay in stool specimens from children with diarrhea J Med Virol 2004 72 496 501 10.1002/jmv.20009 14748075

[B18] Konkel ME Gray SA Kim BJ Garvis SG Yoon J Identification of the enteropathogens campylobacter jejuni and campylobacter coli based on the cadF virulence gene and its product J Clin Microbiol 1999 37 510 517 9986804 10.1128/jcm.37.3.510-517.1999 PMC84446

[B19] Stacy-Phipps S Mecca JJ Weiss JB Multiplex PCR assay and simple preparation method for stool specimens detect enterotoxigenic escherichia coli DNA during course of infection J Clin Microbiol 1995 33 1054 1059 7615704 10.1128/jcm.33.5.1054-1059.1995 PMC228103

[B20] Victor T Du Toit R Van Zyl J Bester AJ Van Helden PD Improved method for the routine identification of toxigenic escherichia coli by DNA amplification of a conserved region of the heat-labile toxin a subunit J Clin Microbiol 1991 29 158 161 1993750 10.1128/jcm.29.1.158-161.1991 PMC269721

[B21] Beaudry M Zhu C Fairbrother JM Harel J Genotypic and phenotypic characterization of escherichia coli isolates from dogs manifesting attaching and effacing lesions J Clin Microbiol 1996 34 144 148 8748291 10.1128/jcm.34.1.144-148.1996 PMC228748

[B22] Gunzburg ST Tornieporth NG Riley LW Identification of enteropathogenic Escherichia coli by PCR-based detection of the bundle-forming pilus gene J Clin Microbiol 1995 33 1375 1377 7615759 10.1128/jcm.33.5.1375-1377.1995 PMC228170

[B23] Sethabutr O Venkatesan M Yam S Pang LW Smoak BL Sang WK Echeverria P Taylor DN Isenbarger DW Detection of PCR products of the ipaH gene from shigella and enteroinvasive escherichia coli by enzyme linked immunosorbent assay Diagn Microbiol Infect Dis 2000 37 11 16 10.1016/S0732-8893(00)00122-X 10794934

[B24] Haque R Roy S Siddique A Mondal U Rahman SMM Mondal D Houpt E Petri WA Multiplex real-time PCR assay for detection of Entamoeba histolytica, Giardia intestinalis, and Cryptosporidium spp Am J Trop Med Hyg 2007 76 713 717 17426176

[B25] Van Doornum GJ Guldemeester J Osterhaus AD Niesters HG Diagnosing herpesvirus infections by real-time amplification and rapid culture J Clin Microbiol 2003 41 576 580 10.1128/JCM.41.2.576-580.2003 12574249 PMC149665

